# Optimization of Media Composition to Maximize the Yield of Exopolysaccharides Production by *Lactobacillus rhamnosus* Strains

**DOI:** 10.1007/s12602-019-09581-2

**Published:** 2019-08-13

**Authors:** Magdalena Oleksy-Sobczak, Elżbieta Klewicka

**Affiliations:** Institute of Fermentation Technology and Microbiology, Faculty of Biotechnology and Food Science, Lodz University of Technology, Wolczanska 171/173, 90-924 Lodz, Poland

**Keywords:** *Lactobacillus rhamnosus*, Exopolysaccharides, Optimization, Medium composition, Statistical analysis

## Abstract

Growth media composition is a critical factor influencing the yield of bacterial exopolysaccharides (EPSs), which have attracted the interest of researchers around the world due to their diverse physicochemical and biological properties. This work presents the optimization of media for EPS synthesis by three *Lactobacillus rhamnosus* strains, namely ŁOCK 0943, ŁOCK 0935, and OM-1. The optimized media led to a more than 13-fold increase in EPS yield for *L. rhamnosus* ŁOCK 0943 (from 85 to 1138.2 mg/L), an almost 9-fold increase for *L. rhamnosus* ŁOCK 0935 (from 103.67 to 900 mg/L), and a more than 7-fold increase for *L. rhamnosus* OM-1 (from 133.67 to 987.84 mg/L) as compared to cultures in standard MRS medium (de Man, Rogosa, and Sharpe). It has been found that the main medium-related determinant of EPS synthesis by the studied *L. rhamnosus* strains are the carbon source—in this case, it was fructose and sucrose.

## Introduction

Lactic acid bacteria (LAB), including probiotics can synthesize extracellular polymers known as exopolysaccharides (EPSs), which can be synthesized as well-defined capsules or loosely organized slime. The latter is of greater practical importance as they are used as natural thickeners in the production of dairy products. LAB added to milk synthesize EPSs in situ, naturally improving the rheology of the end products [1–3].

Embracing healthy lifestyles, including healthy nutrition, has become a significant and still expanding worldwide trend affecting consumer attitudes, with the organic food market growing at a steady rate—an estimated 16% annually until 2020 [4]. Therefore, researchers continually seek new natural food additives. In this context, it should be remembered that in addition to the technological properties, EPSs also exhibit certain health benefits. Studies from different research group have shown that they bind heavy metals, exhibit antioxidant properties, lower blood cholesterol levels, and may also be applied as antiviral and anticancer agents [5–7]. Similarly to LAB, the most of EPSs produced by them have gained the GRAS (generally recognized as a safe) status, which makes them even more attractive for both researchers and manufacturers. Also, some EPSs exhibit bifidogenic properties, which means that they enhance the natural human gut microbiota, thus improving host immunity, intestinal peristalsis, and bowel movement, as well as general well-being [8]. EPSs synthesized by *Lactobacillus* spp. can be used as a substrate for more intensive growth by *Bifidobacterium* spp. This is due to the fact that some *Bifidobacterium* strains possess extracellular enzymes belonging to glycoside hydrolase families 2, 13, 36, and 42, and an enzyme active toward glucooligosaccharides [1, 9].

Despite the numerous beneficial properties of LAB EPSs, their yields are still insufficient. It is known that the main determinants of EPS synthesis are culture conditions: growth medium composition, temperature, pH, duration, and so on. Another critical issue is the method of EPS extraction from the medium [5, 10, 11]. However, the first and foremost factor is growth medium composition as it directly affects EPS yield and chemical composition. LAB can produce EPSs with widely varying structures, depending on the strain as well as on the carbon source in the medium. If the EPSs are to be of industrial value, stable culture conditions must be ensured to generate consistent EPS structures with the same, proven biological properties.

One of the major criteria for designing microbiological media is the economic aspect [12]. Growth medium optimization is very costly and time-consuming, and so the first step in the process should involve statistical methods, such as a Plackett–Burman design, which is a handy method of identifying the determinants of various processes. Within a Plackett–Burman matrix, an experimental setup with seven variables requires as few as eight runs (test variants), while a typical setup would require 128 variants. While this statistical analysis does not consider the interactions, which may occur between the variables, this design is beneficial, especially if little is known about a given process beforehand [13, 14].

The objective of the present study was to optimize a growth medium for the efficient and stable synthesis of EPSs by *L. rhamnosus.* This work presents an optimization procedure employing a design of experiment (DOE) statistical tools.

## Materials and Methods

### Bacterial Strains and Culture Conditions

The study involved the following three strains with probiotic properties: *L. rhamnosus* ŁOCK 0943 (GenBank accession nos. KY576902), *L. rhamnosus* ŁOCK 0935 (GenBank accession nos. KY576901), and *L. rhamnosus* OM-1 (GenBank accession nos. KY576903). The tested strains were obtained from the ŁOCK 105 collection (Lodz, Poland). The probiotic properties of the tested *L. rhamnosus* strains have been confirmed in the grant no. 6 P04B 016 13 “Studies on the properties of *Lactobacillus* and *Bifidobacterium* strains determining their probiotic activity” that was financially supported by the State Committee for Scientific Research (Warsaw, Poland). Stock cultures of these strains were grown in MRS (de Man, Rogosa, and Sharpe, Merck, German) broth with 20% v/v glycerol for long-term frozen storage (− 20 °C). The strains were activated by transfer to fresh MRS broth medium and cultivation at 37 °C for 24 h in MRS broth. Before analysis, the strains were pre-cultured in MRS broth under the same incubation conditions.

### Optimization for EPS Production

Preliminary screening for maximum EPS production was carried out by investigating the effects of different carbon and nitrogen sources, and Tween 80, which were tested in one-factor- and multi-factor-at-a-time experiments.

Carbohydrates (glucose, maltose, galactose, sucrose, fructose, and lactose) used in the studies were preselected using the commercially available API biochemical tests (API 50 CHL, bioMérieux SA, Chemin de l’Orme, 69280 Marcy-l’Étoile, France). The concentration of each saccharide in the medium was 20 g/L. The generation of Maillard compounds during sterilization was avoided as the saccharide solutions were subjected to separate sterilization. MRS (without carbohydrates, BTL, Poland) broths were prepared without glucose or sucrose solutions to ensure appropriate final concentrations of the individual components of the growth medium. Furthermore, experiments tested the effects of nitrogen sources, such as yeast extract, meat extract, and peptone K, as well as Tween 80 at the following concentrations 4 g/L, 8 g/L, 10 g/L, and 1 g/L. The constant components of the growth medium were the mineral compounds: potassium hydrogen phosphate (2 g/L), sodium acetate (5 g/L), diammonium hydrogen citrate (2 g/L), magnesium sulfate (0.2 g/L), and manganese(II) sulfate (0.05 g/L).

At the initial stage of the study, a Plackett–Burman experimental design was used to optimize culture conditions for EPS production. Plackett–Burman designs are used to identify the most critical factors early in the experimentation phase when complete knowledge about the system is usually unavailable [15]. The effects of seven variables were studied at two levels, names high, denoted by (1), and low, denoted by (− 1).

In order to optimize saccharide concentrations, a triangular-based DOE for mixtures was used. In developing a matrix for analysis, it should be remembered that the sum of components must equal 1 for any given variant (all components must add up to 100%, and the shares of the various components must not be negative). A saccharide concentration of 100 g/L was adopted as 100%.

### Isolation and Quantification of Exopolysaccharides

A modified MRS medium was inoculated with bacteria at a concentration of 10% (v/v) and cultivated at 37 °C for 72 h. The cultures were heated at 100 °C for 15 min (to inactivate enzymes), and bacterial cells were removed by centrifugation (15 min, 14,534×*g*, 4 °C). Two volumes of cold 96% ethanol were then added to the supernatant to precipitate the EPSs. The mixtures were left for 24 h at 4 °C. The EPSs were collected by centrifugation at 11,772×*g*, for 20 min at 4 °C and dissolved in 10 mL of distilled water. EPSs were purified by dissolving in 15% (w/v) trichloroacetic acid (TCA), and the precipitates were removed by centrifugation (20,000×*g*, 10 min, 4 °C).

The total sugar content in the samples was determined by the modified phenol-sulfuric acid method with glucose used as a standard [16]. First, 1 mL of the sample was mixed with 0.5 mL of 5% aqueous phenol solution (5 g/100 mL of distilled water). After adding 2.5 mL of 95% sulfuric acid (VI), the samples were incubated at 20 ± 2 °C for 10 min, and then each was stirred for 30 s. The mixed samples were incubated for 20 min in a 25 °C water bath. Subsequent measurements of absorbance at wavelength *λ* = 490 nm were performed. The control sample consisted of distilled water (1 mL). All analyses were performed in three independent replicates. The results are shown as the arithmetic means of three repetitions with standard deviations.

### Statistical Analysis

All analyses were performed in three independent replicates. The results are shown as the arithmetic means of three repetitions with standard deviations. In order to determine the statistical significance of differences between the results, ANOVA was performed at *p* = 0.05 using Origin Pro 2017 software.

The effects of individual variables on EPS synthesis were calculated according to Eq. (1), using a Plackett–Burman design:

1 $$ E\left({X}_i\right)=2\left(\sum {M}_i^{+}-{M}_i^{-}\right)/N $$

where *E*(*X*_*i*_) is the effect of the tested variable (*X*_*i*_) and ${M}_i^{+}$ and ${M}_i^{-}$ are responses (EPS synthesis) from trials in which the variable is at high or low levels, respectively. *N* is the total number of trials. Statistical analysis was performed using Statistica ver. 10 software.

In the design of experiments for mixtures, the following quadratic model was adopted:

$$ y=b0\ast x1+b1\ast x2+b2\ast x3+b3\ast x1\ast x2+b4\ast x1\ast x3+b5\ast x2\ast x3 $$

The analysis was made using a simplex-centroid design with additional interior points (Table 1).

**Table 1:** The matrix used in the design for mixtures

	A	B	C
Simplex-centroid design	1	0	0
	1/2	1/2	0
	1/2	0	1/2
	0	1	0
	0	1/2	1/2
	0	0	1
Additional interior points	1/3	1/3	1/3
	2/3	1/6	1/6
	1/6	2/3	1/6
	1/6	1/6	2/3

All mathematical calculations and triangular coordinate charts were made using Statistica ver. 10 software.

## Results

**Fig. 1:**
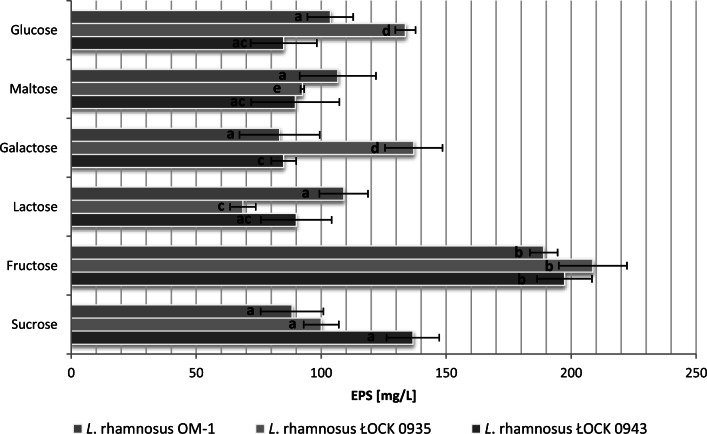
Effects of various carbon sources at a concentration of 20 g/L on EPS production by *L. rhamnosus*. a, b, c, d, e—statistically significant differences between samples of the same strains grown on different carbon sources, *p* ≤ 0.05

In the first step, the effects of different carbon sources on EPS yield from the tested *L. rhamnosus* strains were assessed. The highest yield for all the strains was observed in trials with fructose (Fig. 1). Test results were evaluated in terms of EPS yield, availability of saccharides, and economic aspects. As a result, 4 carbon sources per strain were selected for further study (fructose, glucose, lactose, and sucrose for *L. rhamnosus* ŁOCK 0943 and *L. rhamnosus* OM-1, and fructose, glucose, galactose, and sucrose for *L. rhamnosus* ŁOCK 0935).

**Fig. 2:**
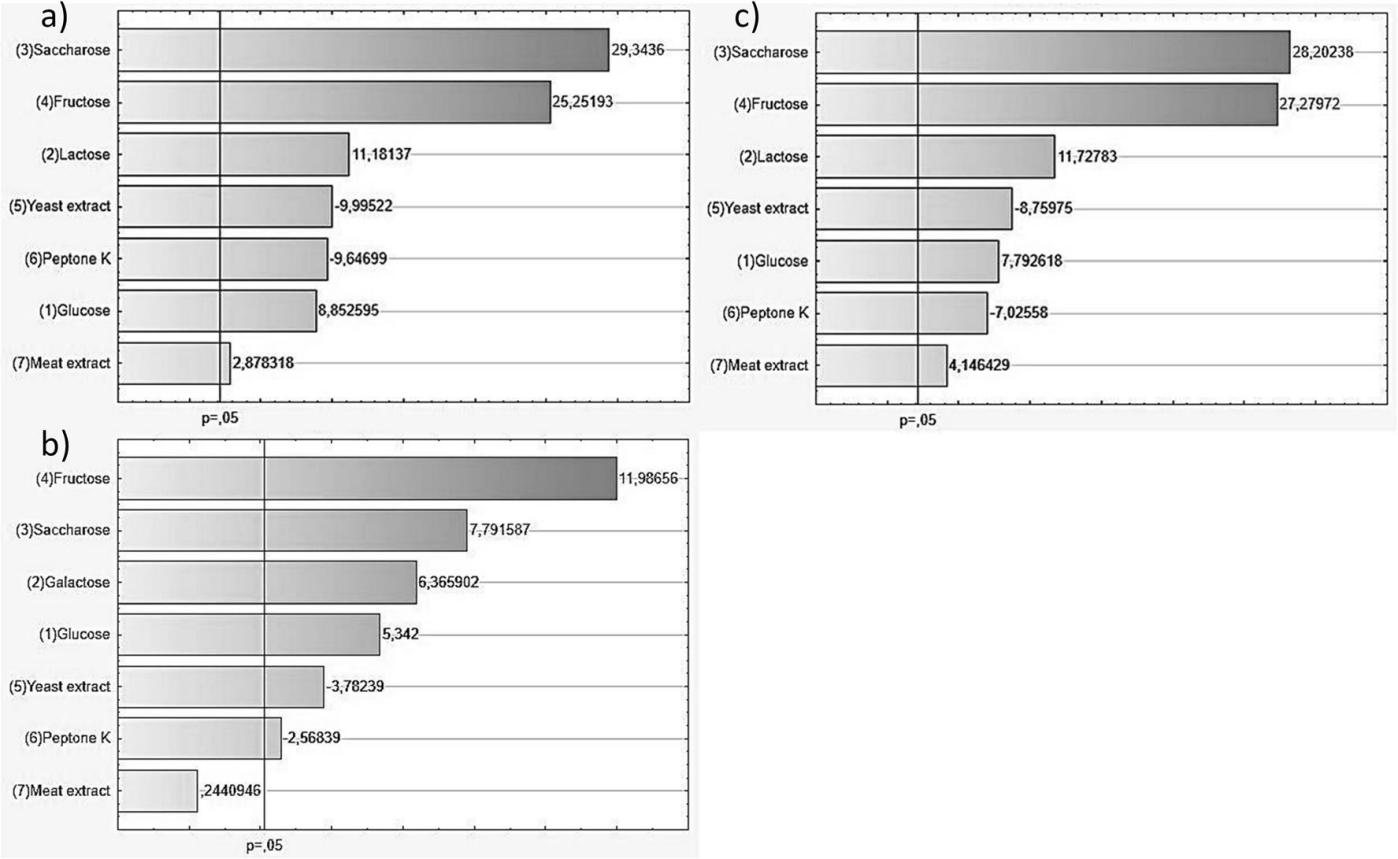
Pareto chart showing a ranking of the variables investigated in the Plackett–Burman design for **a***L. rhamnosus* ŁOCK 0943, **b***L. rhamnosus* ŁOCK 0935, and **c***L. rhamnosus* OM-1

In the next step, the effects of the various medium components on EPS yield were examined using a Plackett–Burman elimination design. Seven factors (4 carbon sources and 3 nitrogen sources) were examined in 8 runs, as shown in the matrix (Table 2). Carbon sources were preselected in a previous step (Fig. 1), while nitrogen sources included yeast extract, peptone K, and meat extract present in the commercially available medium MRS (de Man, Rogosa and Sharpe, Merck, Germany) for LAB culture. Based on EPS yield results, Pareto charts were made for each run using Statistica ver. 10 software (Fig. 2).

**Table 2:** Variables showing the growth medium components used in the Plackett–Burman design and exopolysaccharide yields for the three *L. rhamnosus* strains

No.	Glucose	Lactose/galactose*	Sucrose	Fructose	Yeast extract	Peptone K	Meat extract	EPS (mg/L)		
								ŁOCK 0943	ŁOCK 0935	OM-1
1	− 1	− 1	− 1	1	1	1	− 1	89.0	151.7	135.7
2	1	− 1	− 1	− 1	− 1	1	1	35.0	50.7	37.0
3	− 1	1	− 1	− 1	1	− 1	1	65.3	43.3	70.0
4	1	1	− 1	1	− 1	− 1	− 1	696.7	848.3	665.0
5	− 1	− 1	1	1	− 1	− 1	1	883.3	706.7	857.3
6	1	− 1	1	− 1	1	− 1	− 1	435.0	295.0	371.7
7	− 1	1	1	− 1	− 1	1	− 1	476.0	381.3	456.7
8	1	1	1	1	1	1	1	889.3	913.8	913.3
	− 1 = 0 g/L 1 = 20 g/L				− 1 = 0 g/L 1 = 4 g/L	− 1 = 0 g/L 1 = 10 g/L	− 1 = 0 g/L 1 = 8 g/L			

*Lactose was used for *L. rhamnosus* ŁOCK 0943 and *L. rhamnosus* OM-1; galactose was used for *L. rhamnosus* ŁOCK 0935

Statistical analysis showed that the primary determinant of EPS yields from *L. rhamnosus* was the carbon source in the medium; optimal results for the three studied strains were obtained with fructose and sucrose (Fig. 2).

**Fig. 3:**
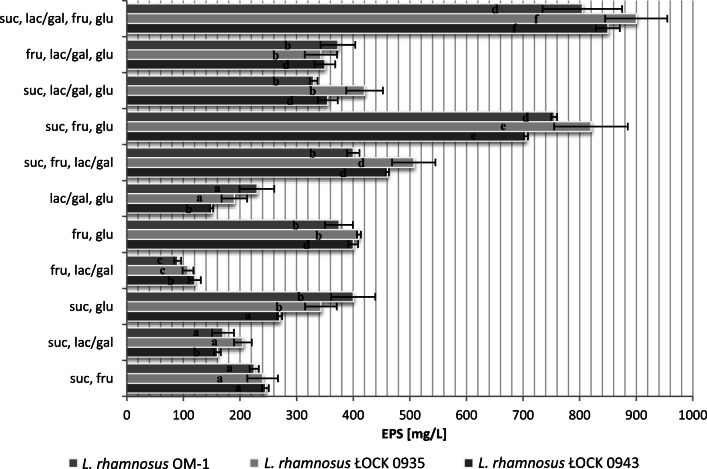
The influence of various carbon sources at a concentration of 20 g/L in multi-factor-at-a-time experiments on the production of exopolysaccharides by *L. rhamnosus*. suc, sucrose; fru, fructose; glu, glucose, lac/gal, lactose for *L. rhamnosus* ŁOCK 0943 and *L. rhamnosus* OM-1 and galactose for *L. rhamnosus* ŁOCK 0935; a, b, c, d, e, f—statistically significant differences between samples of the same strains grown on different carbon sources, *p* ≤ 0.05

Although Plackett–Burman analysis is a very convenient tool in the initial phase of optimization, it does not reflect the interactions that may occur between the various components of the matrix. Thus, the next step of the study addressed the effects of interactions between different carbon sources (at a concentration of 20 g/L each) on EPS yield (Fig. 3). The experiment confirmed the existence of such interactions. The greatest EPS yield was observed in media with sucrose, fructose, glucose, and lactose or galactose as well as in a medium with sucrose, fructose, and glucose. These combinations of saccharides resulted in an approx. 8-fold increase in EPS yield for *L. rhamnosus* ŁOCK 0943 and ŁOCK 0935, and an approx. 6-fold increase for *L. rhamnosus* OM-1. Further analysis involved the variant with sucrose, fructose, and glucose, as it met the economic criterion, while also offering the opportunity to synthesize EPSs with complex structures.

Once the determinants affecting EPS synthesis by *L. rhamnosus* had been enabled, and carbon sources had been selected, the next step aimed to optimize saccharide concentrations in the medium. For this purpose, another DOE for mixtures was performed using Statistica ver. 10. An extended matrix was used to increase the reliability of results (Table 3).

**Table 3:** Design of experiments for mixtures with additional interior points

No.	Fructose	Glucose	Sucrose	*L. rhamnosus* ŁOCK 0943		*L. rhamnosus* ŁOCK 0935		*L. rhamnosus* OM-1	
				EPS (mg/L)	SD	EPS (mg/L)	SD	EPS (mg/L)	SD
1	1	0	0	806.15	2.00	802.00	2.96	789.16	12.14
2	1/2	1/2	0	793.92	10.64	770.92	14.00	771.96	16.45
3	1/2	0	1/2	777.55	21.71	778.69	12.93	791.85	8.74
4	0	1	0	550.36	41.26	429.77	22.87	624.12	41.51
5	0	1/2	1/2	780.87	12.47	764.39	7.03	791.43	2.85
6	0	0	1	801.90	3.76	780.87	16.15	795.99	2.49
7	1/3	1/3	1/3	794.75	5.58	781.18	11.41	794.54	7.03
8	2/3	1/6	1/6	777.76	1.12	797.03	0.31	786.88	2.51
9	1/6	2/3	1/6	768.02	2.18	775.27	13.80	776.83	10.64
10	1/6	1/6	2/3	790.71	12.28	786.46	18.81	798.48	1.90
11*	1/2	1/2	1/2	802.62	1.24	805.52	4.76	794.02	11.22
12*	1/5	1/5	1/5	769.57	7.17	775.07	5.49	767.92	1.26

1—100g/L; 1/2—50 g/L; 1/5—20 g/L; 1/3—33 g/L; 2/3—66 g/L; 1/6—17 g/L. *Variants not used in DOE statistical analysis—mixture designs and triangular surfaces

Based on the knowledge about the process gained from the previous experiments, additional two variants were developed for the design, but they were not used in statistical analysis, because the sum of the components did not equal 1 (100%), which is a prerequisite for DOE for mixtures. However, those additional variants constituted an important comparative reference for the other variants. Based on the prior analysis, value 1 was defined as a concentration of 100 g/L. The highest EPS yield was obtained with a medium containing fructose (variant 1) for *L. rhamnosus* ŁOCK 0943, with glucose, fructose, and sucrose (variant 11) for *L. rhamnosus* ŁOCK 0935, and with sucrose (variant 6) for *L. rhamnosus* OM-1.

**Fig. 4:**
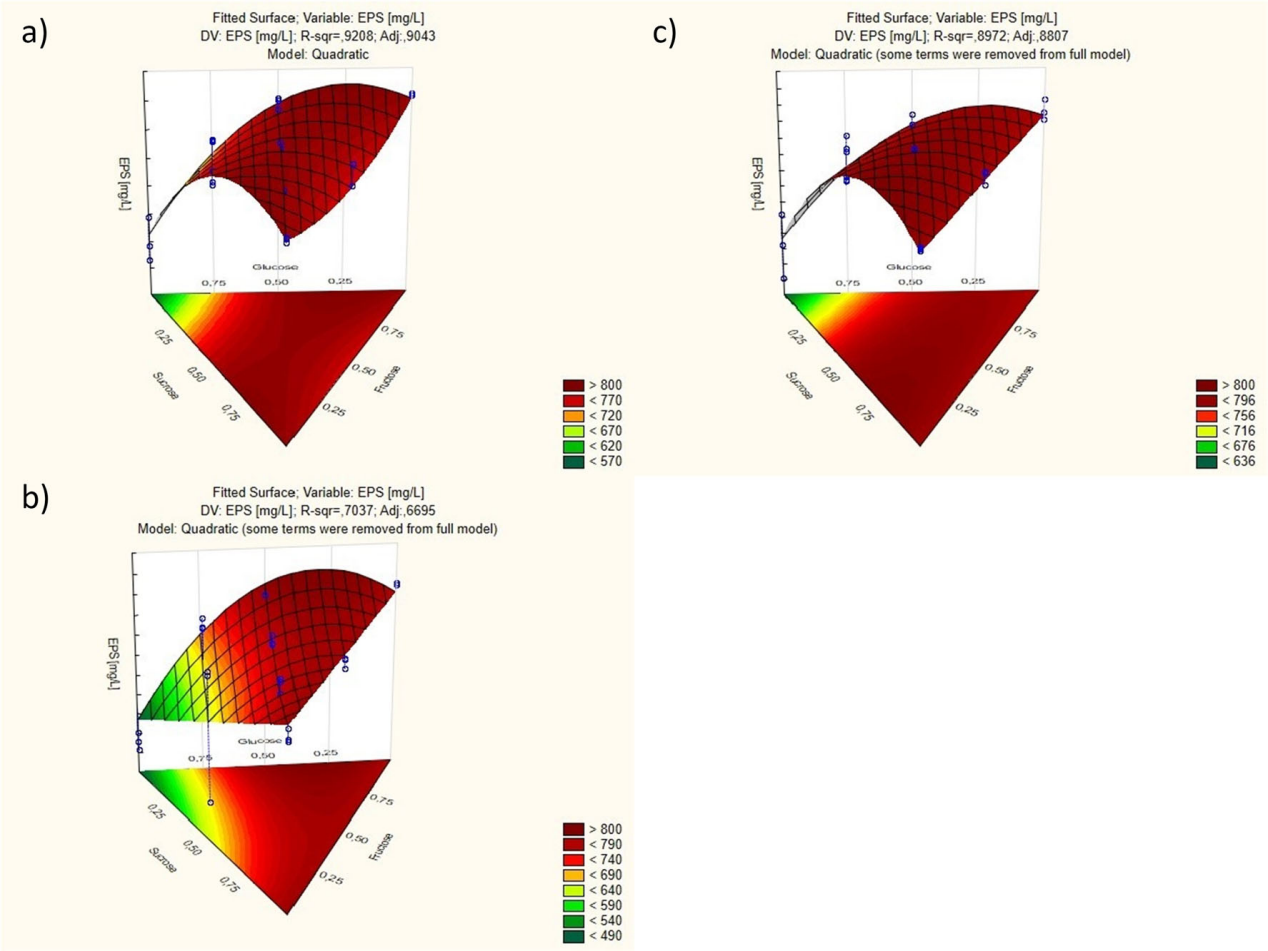
Results charts of experiments for mixtures for **a***L. rhamnosus* ŁOCK 0943, **b***L. rhamnosus* ŁOCK 0935, and **c***L. rhamnosus* OM-1

The results were used to plot a chart, which was a triangle in a three-dimensional space (Fig. 4). Only the points inside the triangle are valid mixtures. The charts show areas with the optimal concentrations of the various saccharides for EPS yield (value > 800). However, analysis of all results revealed no statistically significant differences between the media offering the highest results and the medium with a lower saccharide concentration. Thus, considering the economic aspect, further studies focused on variant 11, which contained fructose, glucose, and sucrose at a concentration of 20 g/L.

The last step of medium composition optimization for EPS synthesis involved analysis of the effects of nitrogen compounds and Tween 80, the latter providing a source of fatty acids necessary for LAB metabolism. The constant components were the mineral salts present in commercially available MRS (see “Isolation and Quantification of Exopolysaccharides” section) and saccharides (fructose, glucose, sucrose) at a concentration of 20 g/L.

**Fig. 5:**
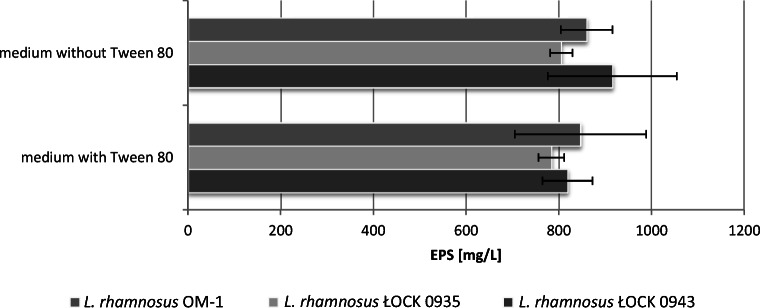
Effects of Tween 80 on exopolysaccharide production by the tested *L. rhamnosus* strains

Analysis of results did not reveal any statistically significant differences in EPS yield between media with and without Tween 80, a nonionic substance, for all the studied strains (Fig. 5). However, since in addition to supplying fatty acids to LAB, Tween 80 also prevents cell aggregation, it was not removed from the optimized growth medium for EPS synthesis.

**Fig. 6:**
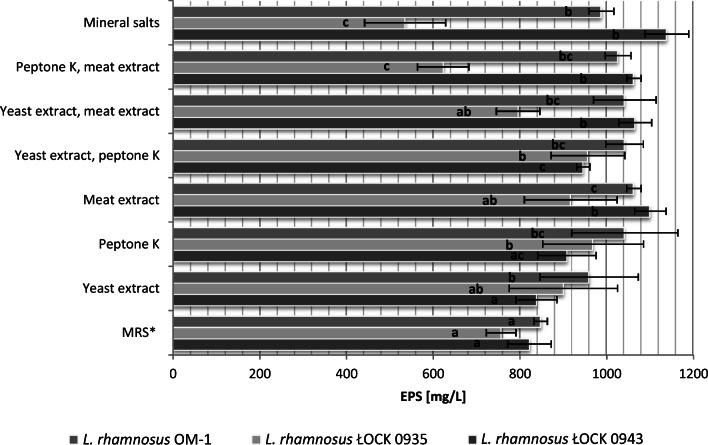
The influence of nitrogen sources on the production of exopolysaccharides by *L. rhamnosus*. *Commercially available MRS without glucose (BTL, Poland) with added fructose, sucrose, and glucose; a, b, c—statistically significant differences between samples of the same strains, *p* ≤ 0.05

The results clearly show that growth medium modeling with nitrogen compounds influences EPS yields (Fig. 6). The results for *L. rhamnosus* ŁOCK 0943 and *L. rhamnosus* OM-1 showed maximum EPS production for media with only mineral salts present. As compared to data from the first experiment (Fig. 1), the application of media containing only saccharides and mineral salts enabled an approx. 5-fold increase in EPS production. In the case of *L. rhamnosus* ŁOCK 0935, the best results were obtained for media containing peptone K as well as yeast extracts with peptone K. However, statistical analysis did not reveal significant differences between these two cultures and the culture conducted in the medium with yeast extract. Since yeast extract is a cheaper source of amino acids and peptides than peptone K, media containing it are more attractive with a view to industrial-scale EPS production by lactobacilli in the future.

Based on the presented results, an optimized growth medium was developed for efficient and stable EPS synthesis by the studied strains (Table 4).

**Table 4:** Growth medium optimized for efficient synthesis of exopolysaccharides by the tested *Lactobacillus* strains

Component	*L. rhamnosus* ŁOCK 0943	*L. rhamnosus* ŁOCK 0935	*L. rhamnosus* OM-1
Mineral salts	K_2_HPO_4_ (2 g/L), CH_3_COONa (5 g/L), C6H14N2O7 (2 g/L), MgSO_4_ (0.2 g/L), and MnSO_4_ (0.05 g/L)		
Carbon source	Fructose, glucose, and sucrose at a concentration of 20 g/L		
Nitrogen source	-	Yeast extract (4 g/L)	-
Tween 80	Concentration of 1 g/L		

- absent

## Discussion

This work focuses on three *L. rhamnosus* strains with the highest EPS production potential selected from a total of 60 LAB strains by previous research [17]. Indeed, according to the literature, *L. rhamnosus* offer the highest yields of EPSs, for example, *L. rhamnosus* RW-9595 M cultured in a whey medium produced up to approx. 2.7 g/L EPS [5, 18]. However, whey has a highly variable chemical composition, which may result in the synthesis of EPSs with different structures, and thus inconsistent biological and technological properties. Therefore, the objective of this work was to develop an inexpensive growth medium for the efficient synthesis of structurally consistent EPSs.

In the first step, the determinants increasing EPS yield were identified to be fructose and sucrose. Similar results were reported for *L. plantarum* NTMI05 and NTMI20, but in that case, the highest EPS yield (approx. 300 mg/L) was obtained in a medium containing glucose (20 g/L) [19]. Higher EPS yields in media with glucose as the only carbon source were also observed for *L. pentosus* LPS26 and for *L. delbrueckii* subsp. *bulgaricus* CNRZ 118 and CNRZ 416 [20, 21]. In turn, lactose was successfully used for high-yield EPS production by *Lactobacillus helveticus* MB2-1 [22]. In contrast, researchers, who also used the Plackett–Burman matrix to optimize growth medium for another LAB strain, *L. rhamnosus* E/N, identified yeast extract to be the main EPS yield determinant, followed by saccharide type (galactose) [23].

The optimization of a growth medium is a very complex process which should consider interactions that may occur between its components. The current study clearly shows that the type of mixture used has a significant effect on EPS production by the studied LAB strains. A triangular-based design of experiments for mixtures revealed that application of fructose, glucose, and sucrose at a concentration of 20 g/L each in the medium is optimal. Studies conducted by Cerning et al. indicate that both the yield and the composition of the synthesized EPS depend on the carbon source present in the medium [24]. Therefore, it should also be noted that the application of 3 different saccharides may result in EPSs with more complex structural characteristics than is the case with media containing one carbon source only.

In the next step, optimization was performed for nitrogenous compounds (yeast extract, meat extract, peptone K) and Tween 80. It was shown that while Tween 80 did not directly affect EPS production, it was nevertheless an important component preventing cell aggregation, and as such, it was not removed from the formula. In contrast, in a similar study, Tween 80 was found to hurt EPS synthesis by *L. acidophilus* [25]. In the present experiments, the best EPS yields from *L. rhamnosus* ŁOCK 0943 and OM-1 were identified for media without nitrogenous components, containing only mineral salts and saccharides. In the optimized growth medium, *L. rhamnosus* ŁOCK 0943 gave an EPS yield of 1138.2 mg/L, while *L. rhamnosus* ŁOCK OM-1 produced 987.84 mg/L. In the case of *L. rhamnosus* ŁOCK 0935, the optimum results were found in a medium with yeast extract as a nitrogen source, resulting in an EPS yield of 900 mg/L. Much higher results were reported for *L. acidophilus* with the optimized medium containing 0.6 mL of Tween 80, 3.6 g of dipotassium hydrogen phosphate, and 4.1 g of trisodium citrate (at a culture volume of 1 L) [25]. Under such conditions, EPS yield from mutant *L. acidophilus* strains reached 3.96 ± 0.08 g/L. The strains were created by chemical mutation using 0.2% diethyl sulfate. The present authors avoided mutants as they may cause genome instability, undermining the quality and effectiveness of the synthesized EPSs. Moreover, genetically modified strains are generally avoided in the industry. In the study on EPSs from *L. confusus* TISTR 1948, a medium with sucrose was used under high salinity stress [26]. It was found that under optimized culture conditions with 4.97% of NaCl and sucrose content of 136.5 g/L, EPS yield was 2.5 times higher (86.36 g/L) than in modified MRS containing only 120 g/L sucrose without NaCl (33.4 g/L of EPS). One of the reasons why bacteria synthesize EPSs is cell protection from adverse environmental conditions. As EPSs have considerable water retention capacity, the addition of NaCl may have accelerated *L. confusus* metabolism to produce more EPSs.

The medium developed by the authors for the effective synthesis of exopolysaccharides is more than four times cheaper than the standard MRS medium and is easy to prepare. It is also important that the organic nitrogen source has been removed from the medium (or strongly limited). The process of exopolysaccharides synthesis generates a large amount of protein laden wastewater. By limiting the amount of protein in the medium, the costs of wastewater utilization were reduced. This is beneficial for the protection of the natural environment. Thanks to these features, it can become a medium commonly used for the production of lactobacilli exopolysaccharides on an industrial scale.

## Conclusions

In summary, *L. rhamnosus* ŁOCK 0943, ŁOCK 0935, and OM-1 have a considerable application potential to produce extracellular biopolymers. The presented experiments enabled the optimization of growth medium for exopolysaccharides synthesis by the studied strains. Their culture in this medium led to an approx. 13-fold, 9-fold, and 7-fold increase in EPS production at much lower costs as compared to culture in the standard MRS medium for *L. rhamnosus* ŁOCK 0943, ŁOCK 0935, and OM-1, respectively. From the technological point of view, the developed growth medium is inexpensive and easy to prepare and its use significantly reduces the amount of wastewater loaded with protein. The present authors are planning to conduct further studies into other culture parameters to additionally enhance EPS yield. Despite international research efforts on a variety of strains, there is still considerable room for improvement in optimizing culture conditions for LAB synthesizing industry-grade EPSs.

## References

[1] Ruas-Madiedo P, Hugenholtz J, Zoon P (2002). An overview of the functionality of exopolysaccharides produced by lactic acid bacteria. Int Dairy J.

[2] Salazar N, Prieto A, Leal JA, Mayo B, Bada-Gancedo JC, de los Reyes-Gavilán CG, Ruas-Madiedo P (2009). Production of exopolysaccharides by *Lactobacillus* and *Bifidobacterium* strains of human origin, and metabolic activity of the producing bacteria in milk. J Dairy Sci.

[3] Yilmaz MT, Dertli E, Toker OS, Tatlisu NB, Sagdic O, Arici M (2015). Effect of *in situ* exopolysaccharide production on physicochemical, rheological, sensory, and microstructural properties of the yogurt drink ayran: an optimization study based on fermentation kinetics. J Dairy Sci.

[4] TechSci Research (2015) Global Organic Food Market Forecast and Opportunities, 2020. https://www.techsciresearch.com/report/global-organic-food-market-forecast-and-opportunities-2020/450.html. Accessed 11 May 2011

[5] Badel S, Bernardi T, Michaud P (2011). New perspectives for Lactobacilli exopolysaccharides. Biotechnol Adv.

[6] El Ghany KA, Hamouda R, Elhafez EA, Mahrous H, Salem-Bekhit M, Hamza HA (2015). A potential role of *Lactobacillus acidophilus* LA1 and its exopolysaccharides on cancer cells in male albino mice. Biotechnol Biotec Eq.

[7] Wang J, Zhao X, Tian Z, Yang Y, Yang Z (2015). Characterization of an exopolysaccharide produced by *Lactobacillus plantarum* YW11 isolated from Tobet Kefir. Carbohydr Polym.

[8] Sarikaya H, Aslim B, Yuksekdag ZN (2016). Assessment of anti-biofilm activity and bifidogenic growth stimulator (BGS) effect of lyophilized exopolysaccharides (l-epss) from Lactobacilli strains. Int J Food Prop.

[9] Widyastuti Y, Rohmatussolihat FA (2014). The role of lactic acid bacteria in milk fermentation. J Food Nutri Sci.

[10] Patel A, Prajapati JB (2013). Food and health applications of exopolysaccharides produced by lactic acid bacteria. J Adv Dairy Res.

[11] Polak-Berecka M, Waśko A, Skrzypek H, Kreft A (2013). Production of exopolysaccharides by a probiotic strain of *Lactobacillus rhamnosus*: biosynthesis and purification methods. Acta Aliment.

[12] Oleksy M, Klewicka E (2018). Exopolysaccharides produced by *Lactobacillus* sp.: biosynthesis and applications. Crit Rev Food Sci.

[13] Naveena BJ, Altaf M, Bhadriah K, Reddy G (2005). Selection of medium components by Plackett–Burman design for production of L (+) lactic acid by *Lactobacillus amylophilus* GV6 in SSF using wheat bran. Bioresour Technol.

[14] Zhou J, Yu X, Ding C, Wang Z, Zhou Q, Pao H, Cai W (2011). Optimization of phenol degradation by *Candida tropicalis* Z-04 using Plackett-Burman design and response surface methodology. J Environ Sci.

[15] Reddy LVA, Wee YJ, Yun JS, Ryu HW (2008). Optimization of alkaline protease production by batch culture of *Bacillus* sp. RKY3 through Plackett–Burman and response surface methodological approaches. Bioresour Technol.

[16] Dubois M, Gilles KA, Hamilton JK, Rebers PAT, Smith F (1956). Colorimetric method for determination of sugars and related substances. Anal Chem.

[17] Oleksy M, Klewicka E (2017). Screening of efficient exopolysaccharide-producing strains of *Lactobacillus* sp. Żywność Nauka Technologia Jakość.

[18] Macedo MG, Lacroix C, Gardner NJ, Champagne CP (2002). Effect of medium supplementation on exopolysaccharide production by *Lactobacillus rhamnosus* RW-9595 M in whey permeate. Int Dairy J.

[19] Imran MYM, Reehana N, Jayaraj KA, Ahamed AAP, Dhanasekaran D, Thajuddin N, Muralitharan G (2016). Statistical optimization of exopolysaccharide production by *Lactobacillus plantarum* NTMI05 and NTMI20. Int J Biol Macromol.

[20] Sánchez JI, Martínez B, Guillén R, Jiménez-Díaz R, Rodríguez A (2006). Culture conditions determine the balance between two different exopolysaccharides produced by *Lactobacillus pentosus* LPS26. Appl Environ Microbiol.

[21] Petry S, Furlan S, Crepeau MJ, Cerning J, Desmazeaud M (2000). Factors affecting exocellular polysaccharide production by *Lactobacillus delbrueckii* subsp. *bulgaricus* grown in a chemically defined medium. Appl Environ Microbiol.

[22] Li W, Ji J, Rui X, Yu J, Tang W, Chen X, Dong M (2014). Production of exopolysaccharides by *Lactobacillus helveticus* MB2-1 and its functional characteristics in vitro. LWT-Food Sci Technol.

[23] Polak-Berecka M, Waśko A, Kubik-Komar A (2014). Optimization of culture conditions for exopolysaccharide production by a probiotic strain of *Lactobacillus rhamnosus* E/N. Pol J Microbiol.

[24] Cerning J, Renard CMGC, Thibault JF, Bouillanne C, Landon M, Desmazeaud M, Topisirovic L (1994). Carbon source requirements for exopolysaccharide production by *Lactobacillus casei* CG11 and partial structure analysis of the polymer. Appl Environ Microbiol.

[25] Liu Q, Huang X, Yang D, Si T, Pan S, Yang F (2016). Yield improvement of exopolysaccharides by screening of the *Lactobacillus acidophilus* ATCC and optimization of the fermentation and extraction conditions. EXCLI J.

[26] Seesuriyachan P, Kuntiya A, Hanmoungjai P, Techapun C, Chaiyaso T, Leksawasdi N (2012). Optimization of exopolysaccharide overproduction by *Lactobacillus confusus* in solid state fermentation under high salinity stress. Biosci Biotechnol Biochem.

